# Targets and Mechanism Used by Cinnamaldehyde, the Main Active Ingredient in Cinnamon, in the Treatment of Breast Cancer

**DOI:** 10.3389/fphar.2020.582719

**Published:** 2020-12-09

**Authors:** Yufei Liu, Tian An, Donggui Wan, Bowen Yu, Yingyi Fan, Xiaohua Pei

**Affiliations:** ^1^ Beijing University of Chinese Medicine Third Affiliated Hospital, Beijing, China; ^2^ Oncology Department of Integrated Traditional Chinese and Western Medicine, China-Japan Friendship Hospital, Beijing, China; ^3^ School of Traditional Chinese Medicine, Beijing University of Traditional Chinese Medicine, Beijing, China; ^4^ Xiamen Hospital, Beijing University of Chinese Medicine, Xiamen, China

**Keywords:** cinnamaldehyde, breast cancer, network pharmacology, active components, MDA-MB-231

## Abstract

**Background:** Breast cancer has become one of the most common malignant tumors in women owing to its increasing incidence each year. Clinical studies have shown that *Cinnamomum cassia* (L.) *J. Presl* (cinnamon) has a positive influence on the prevention and treatment of breast cancer.

**Aim:** We aimed to screen the potential targets of cinnamon in the treatment of breast cancer through network pharmacology and explore its potential therapeutic mechanism through cell experiments.

**Methods:** We used the TCMSP, TCM Database @ Taiwan, and TCMID websites and established the active ingredient and target database of cinnamon. Thereafter, we used the GeneCards and OMIM databases to establish a breast cancer-related target database, which matched the cinnamon target database. Based on the matching results, the STRING database was used to analyze the interaction between the targets, and the biological information annotation database was used to analyze the biological process of the target (gene ontology) and the pathway enrichment of Kyoto Encyclopedia of Genes and Genomes (KEGG). After establishing the layout of the analysis, we used Cytoscape 3.6.0 software for network analysis. Finally, the cell experiment was used to verify the anti-breast cancer effect of cinnamaldehyde.

**Results:** Our research showed that the main components of cinnamon, including cinnamaldehyde, can play a role in the treatment of breast cancer through 59 possible important targets. Subsequently, enrichment analysis by gene ontology and Kyoto Encyclopedia of Genes and Genomes showed that 83 cell biological processes and 37 pathways were associated with breast cancer (*p* < 0.05), including the peroxisome proliferator-activated receptor and PI3K-Akt pathway, which are closely related to tumor cell apoptosis. *In vitro* cell verification experiments showed that cinnamaldehyde can significantly inhibit cell proliferation, change cell morphology, inhibit cell migration and invasion ability, and promote cell apoptosis.

**Conclusion:** Our results showed that cinnamaldehyde is a potential novel drug for the treatment and prevention of breast cancer.

## Introduction

*Cinnamon*, the dried bark of *Cinnamomum cassia* (L.) *J. Presl*, is one of the most commonly used traditional herbs worldwide. Studies have shown that the chemical components of cinnamon exert anti-tumor effects. Cinnamic acid derivatives can inhibit the growth of lung cancer cells (A549), breast cancer cells (MCF-7), and MCF-10A (Reddy et al., 2016). Cinnamon essential oil is cytotoxic *in vitro* and exhibits certain inhibitory effects on PC3, A549, and MCF-7 prostate cancer cells, with PC3 exerting the strongest inhibitory effect (Zu et al., 2010). Trans-cinnamic acid can inhibit melanoma proliferation and tumor growth (Cabello et al., 2009). Eugenol is another important active ingredient in cinnamon. In fact, *in vivo* studies have shown that eugenol can upregulate the expression of p53 and p21WAF1 and promote apoptosis of cancer cells in mice with skin cancer (Kaur et al., 2010). Cinnamaldehyde can also exert a significant anticancer effect on HepG2 hepatoma cells by reducing the expression of the anti-apoptotic protein, Bcl-XL (Ng and Wu, 2011).

Breast cancer is a malignant tumor with a high clinical incidence. Accordingly, approximately 1.39 million new breast cancer patients are identified worldwide each year (Siegel et al., 2018). Its incidence rate is the highest among malignant tumors for women, and its mortality rate ranks second among female malignancies (Bray et al., 2018). In China, the annual growth rate of breast cancer patients has exceeded the world average. According to the 2015 China Breast Cancer Survey, approximately 26,000 new breast cancer patients are diagnosed each year, causing approximately 70,000 deaths each year (Chen et al., 2016). Triple-negative breast cancer (TNBC) is a special type of breast cancer that is characterized by the progesterone receptor (PR), estrogen receptor (ER), and human epidermal growth factor receptor 2 (HER2), all of which are negative (Parise and Caggiano, 2017; Liao et al., 2018). TNBC accounts for 15–20% of all breast cancer pathological types, most of which occur in premenopausal young women, with high malignancy and poor prognosis (Rida et al., 2018). Besides this, 30–40% of TNBC cases can develop metastatic breast cancer, with visceral metastases being more common, especially lung and brain metastases (Foulkes et al., 2010). TNBC is a group of highly heterogeneous mixed breast cancers with seven subtypes, and endocrine and anti-HER2 treatment are ineffective treatment options for this cancer type (Lehmann et al., 2011). Currently, TNBC mainly relies on adjuvant therapy such as chemotherapy. Therefore, there is an urgent need to develop new drugs and targets for the treatment of refractory breast cancer TNBC.

In the current study, we used network pharmacology to predict the main ingredients and potential therapeutic targets that are responsible for the anti-breast cancer effects of cinnamon. Subsequently, based on the results of network analysis, we investigated the gene ontology (GO) terms and pathways of cinnamon and breast cancer, and carried out biological verification using breast cancer cells *in vitro*. Our research findings may provide experimental data for further development and utilization of cinnamon and may serve as a reference for research on traditional herbs for cancer and cancer-related diseases.

## Methods and Materials

### Screening of the Active Ingredients of Cinnamon by Network Pharmacology

We built a network of potential therapeutic targets for cinnamon based on previous research (Lu et al., 2020). Specifically, all ingredients in cinnamon were obtained through three databases: TCM Systems Pharmacology Database and Analysis Platform (TCMSP), TCM Database @ Taiwan (TCM Database @ Taiwan), and the TCM Integrated Database (TCMID). Subsequently, by setting Lipinski rule-based drug-likeness (DL) and oral bioavailability (OB), the active ingredients related to cinnamon were screened (OB ≥ 20%, DL ≥ 0.1).

### Correlation Analysis of Cinnamon and the Breast Cancer-Related Targets

The corresponding target of the compound in cinnamon was verified through the TCMSP database. If no target information for the compound was found on the platform, the small-molecule structure information of the component was searched using the PubChem database and saved in the SMILES format. Thereafter, we used Swiss Target Prediction according to its chemistry similarity to find targets. The UniProt database was used to normalize the gene information and eliminate genes without UniProt ID from human samples. The breast cancer-related targets were obtained through the integration of multi-source databases. Specifically, “breast cancer” was used as the search term; the results obtained from the GeneCards database and Online Mendelian Inheritance in Man (https://omim.org/) search were comprehensively analyzed and a breast cancer target library was constructed. Through Venny 2.1.0 (http://bioinfo.cnb.csic.es/tools/venny/index.html), the targets associated with breast cancer and the main compounds in cinnamon were related for a visual demonstration of the targets that intersect between them.

### Screening of the Main Components and Targets in Cinnamon for the Treatment of Breast Cancer

The intersection of the corresponding target of the active ingredient in cinnamon and the target of breast cancer disease was used to construct a protein–protein interaction (PPI) map in the STRING database. The matched targets were analyzed using the Database for Annotation, Visualization, and Integrated Discovery (https://david.ncifcrf.gov/) v6.8. biological information annotation database for target GO (http://geneontology.org/) biological process analysis and Kyoto Encyclopedia of Genes and Genomes (KEGG, https://www.kegg.jp/) pathway enrichment analysis. *p* < 0.05 was considered statistically significant.

### Cell Culture and Treatment With Cinnamaldehyde

MDA-MB-231, a human breast cancer cell line, was obtained from the Be Na Culture Collection (Biotechnology Research Institute) and cultured with RPMI 1640 (11875093, INVITROGEN) supplemented with 10% fetal bovine serum (FBS, Corning-Cellgro Bio Inc., New Zealand) and 1% penicillin-streptomycin in an incubator set to 37°C and 5% CO_2_. When the confluence of MDA-MB-231 reached 80–90%, it was treated with different concentrations of cinnamaldehyde (CA, 110710-201821; Chemical formula, Supplementary Figure S1) for subsequent detection.

### Effects of Cinnamaldehyde on the Viability of MDA-MB-231 Cells

The MTT assay was used to analyze the effect of different concentrations of cinnamaldehyde on the viability of MDA-MB-231 cells (Buglak et al., 2018). MDA-MB-231 cells with moderate density were seeded in 96-well plates and cultured with complete medium until adherence occurred. Subsequently, different concentrations of cinnamaldehyde (0, 2.5, 5, 10, 20, 40, 80, and 160 μg/ml) were used to treat breast cancer cells for 24 and 48 h. Thereafter, 20 μl of MTT was added to each well, and the plates were incubated for another 4 h at 37vC. Finally, the medium was aspirated, and DMSO (150 μl/well) was added. A multifunctional microplate reader (FLUO star Omega, BMG Labtech, Germany) was employed to measure the optical density (OD) at 490 nm. GraphPad Prism 8.0 software was used to calculate the IC_50_ at 48 h. The cell proliferation inhibition rate was calculated using the following formula [1 − (OD value of drug group/OD value of the control group)] × 100%.

### Effect of Cinnamaldehyde on MDA-MB-231 Cell Morphology

MDA-MB-231 cells (1 × 10^6^ cells/ml) were seeded in 6-well plates, and their morphology was observed under a microscope after treatment with cinnamaldehyde (0, 10, 15, 20 μg/ml) for 48 h. Subsequently, Hoechst 33258 staining was used to observe the effect of different concentrations of cinnamaldehyde on the cytoplasmic morphology of MDA-MB-231 cells (Hagenlocher et al., 2015).

### Effects of Cinnamaldehyde on the Apoptosis of MDA-MB-231 Cells

MDA-MB-231 cells (1 × 10^6^ cells/mL) were seeded in 6-well plates and treated with cinnamaldehyde (0, 10, 15, 20 μg/ml) for 24 h. A cell digestion solution (Beijing Solbio Technology Co., Ltd., article number: 20171024) was then used to prepare a cell suspension. Thereafter, 100 µl of the cell suspension was pipetted into a 1.5-ml Eppendorf tube. Subsequently, according to the instructions of the fluorescein thiocyanate (FITC)-conjugated Annexin-V apoptosis detection kit (Becton, Dickinson and Company, Franklin Lake, New Jersey), the cell suspension and 5 μl of Annexin-V-FITC were mixed with 5 μl of propidium iodide (PI, United States, batch number: 7040932) and incubated for 15 min. A 150-μl volume of the binding buffer was then added to each test tube and analyzed by flow cytometry.

### Effects of Cinnamaldehyde on the Invasion and Migration of MDA-MB-231 Cells

The cell invasion assay was performed using Corning Transwell Chamber and Matrigel according to a previously described method (Guo et al., 2020). Briefly, a serum-free medium was used to hydrate the Matrigel. Thereafter, cells with different concentrations of cinnamaldehyde intervention and serum-free medium culture were placed in the upper chamber. RPMI 1640 medium supplemented with 10% fetal bovine serum was placed in the lower chamber. After 48 h, the cells were fixed with 4% paraformaldehyde for 30 min and stained with 0.1% crystal violet for 10 min. The invasiveness of the cells was then observed under a microscope.

The migration of cells was investigated using a wound-healing assay. MDA-MB-231 cells (1 × 10^6^) from different administration groups were seeded in triplicate in six-well plates and incubated at 37°C for 48 h. Scratches were generated using a 1-ml micropipette tip when the cells reached 100% confluence. Thereafter, the cells were washed twice with PBS and incubated at 37°C in complete medium. Images were captured after 0 and 48 h, and the wound area was calculated.

### Statistical Analysis

Statistical analysis was performed using GraphPad Prism 8 software. All results are presented as mean ± standard deviation (SD). One-way analysis of variance was performed between multiple groups when the homogeneity of variance and normality were met. Otherwise, Dunnett’s T3 and nonparametric tests were conducted between multiple groups. *p* < 0.05 was determined as a statistical difference.

## Results

### Active Compounds of Cinnamon

**FIGURE 1:**
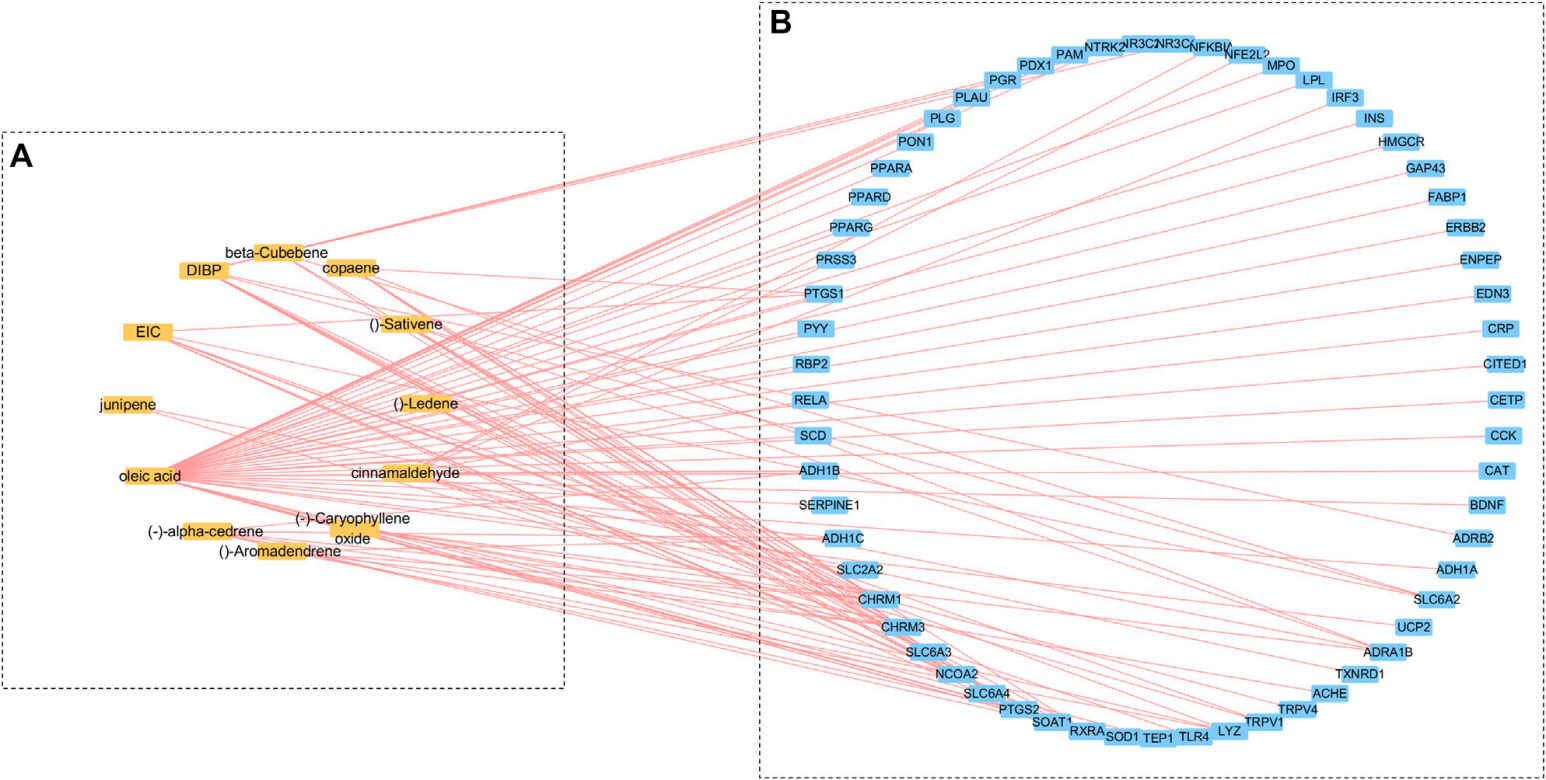
Cinnamon-compounds-breast cancer network. **(A)** Cinnamon-compounds, **(B)** Cinnamon-related compounds-breast cancer. Yellow represents the main compound in cinnamon and blue represents the target associated with breast cancer.

A total of 147 related components of the whole formula was retrieved from three databases, TCMSP, TCM Database @ Taiwan, and TCMID. Based on the ADME thresholds of OB ≥ 20%, DL ≥ 0.1, and Caco-2 > 0, 12 active ingredients were selected. Thereafter, an herb-compounds network was built as shown in Figure 1. Following the construction of the cinnamon-compounds network and an analysis of the 12 active ingredients, the top four ingredients in descending order of edge betweenness were linoleic acid (EIC, MOL000131, OB = 41.9, DL = 0.14, Caco-2 = 1.16), oleic acid (MOL000675, OB = 33.13, DL = 0.14, Caco-2 = 1.17), diisobutyl phthalate (DIBP, MOL000057, OB = 49.63, DL = 0.13, Caco-2 = 0.85), and cinnamaldehyde (CA, MOL000449, OB = 31.99, DL = 0.12, Caco-2 = 1.35) (Figure 1A).

### Prediction and Analysis of the Cinnamon-Related Compounds and Breast Cancer-Related Targets

**FIGURE 2:**
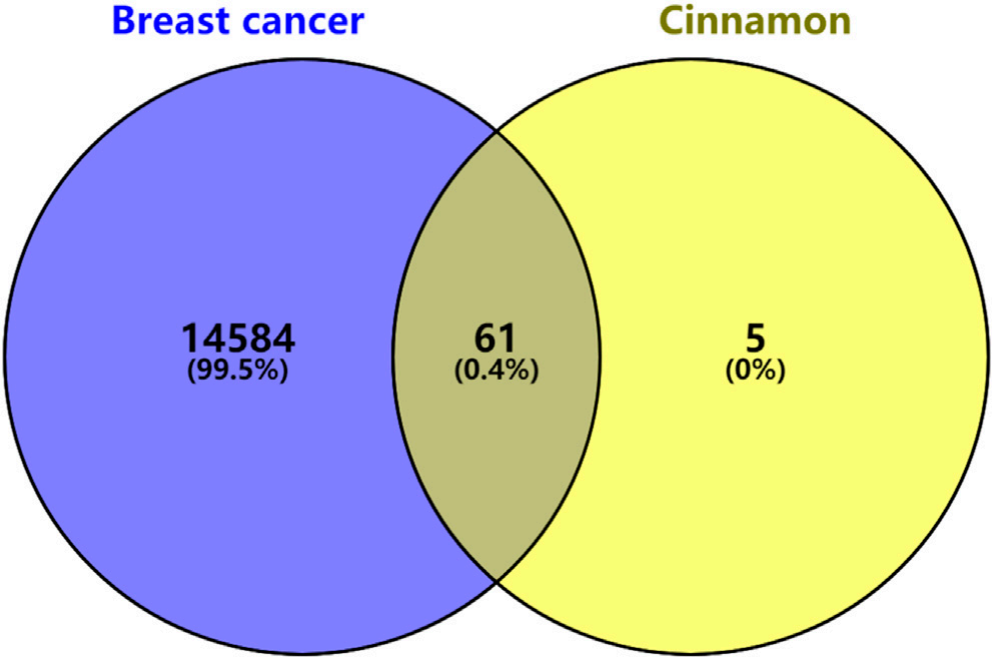
Venn diagram of the targets in breast cancer and cinnamon.

A target fishing analysis was conducted on the 12 cinnamon-related compounds based on chemical similarity. As a result, 66 related targets were obtained. Through the method of integration of multi-source databases, the target data for breast cancer-related targets from GeneCards (13,933) and OMIM (14,301) were integrated. Thereafter, 61 matching targets of cinnamon and breast cancer-related targets were collected as related targets for the anti-breast cancer effects of cinnamon (Figure 2.). Subsequently, we constructed an active ingredient-disease-target network to further screen the effective ingredients responsible for the anti-breast cancer activity of cinnamon (Figure 1B). Among the 12 active ingredients, oleic acid, DIBP, and cinnamaldehyde were identified as the more critical ingredients. Therefore, we speculate that cinnamon may play an anti-breast cancer role mainly through this composition.

### Target Prediction and Analysis

**FIGURE 3:**
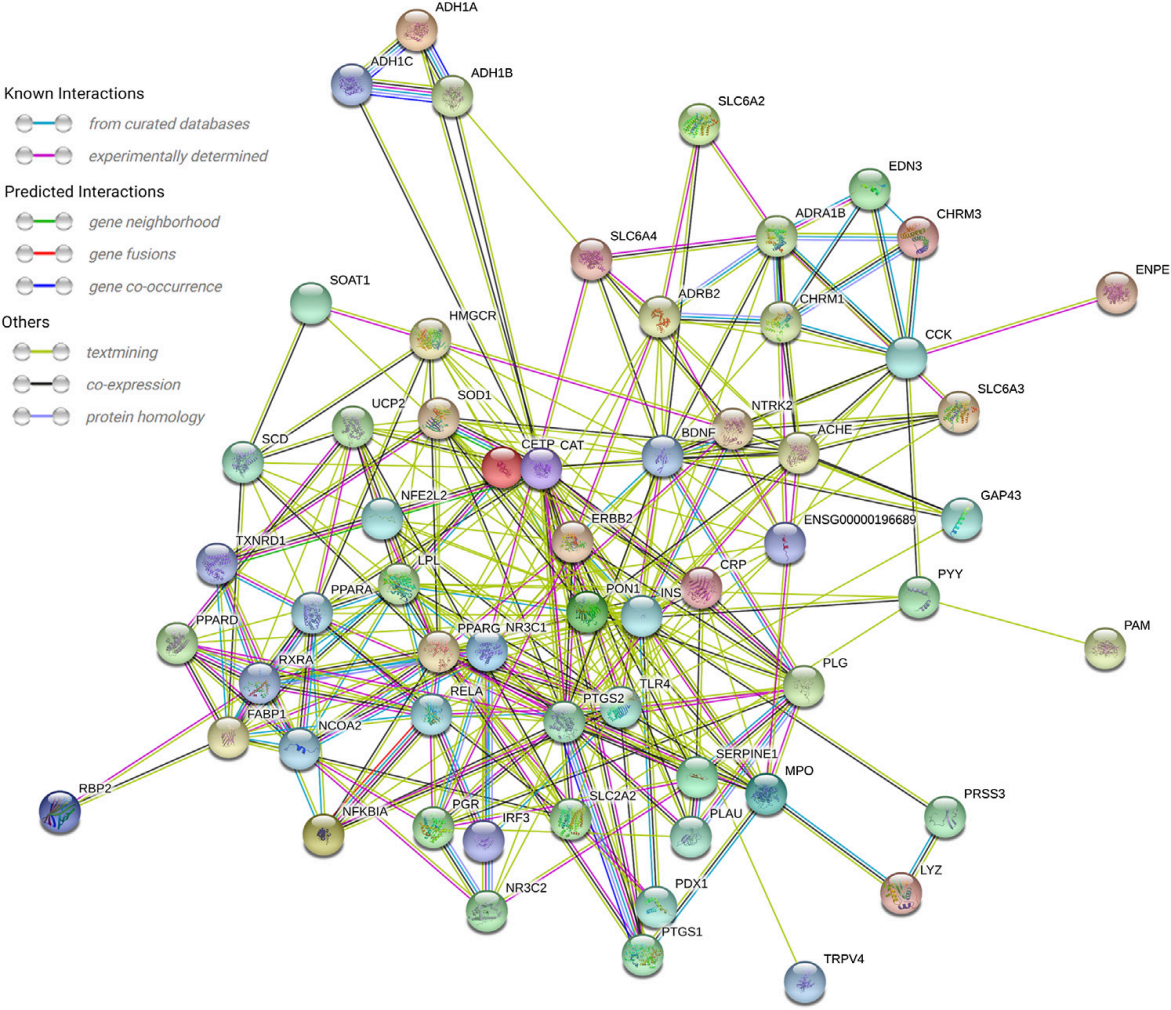
PPI network of 59 nodes. A total of 59 overlapped target genes were used to construct the PPI network.

**FIGURE 4:**
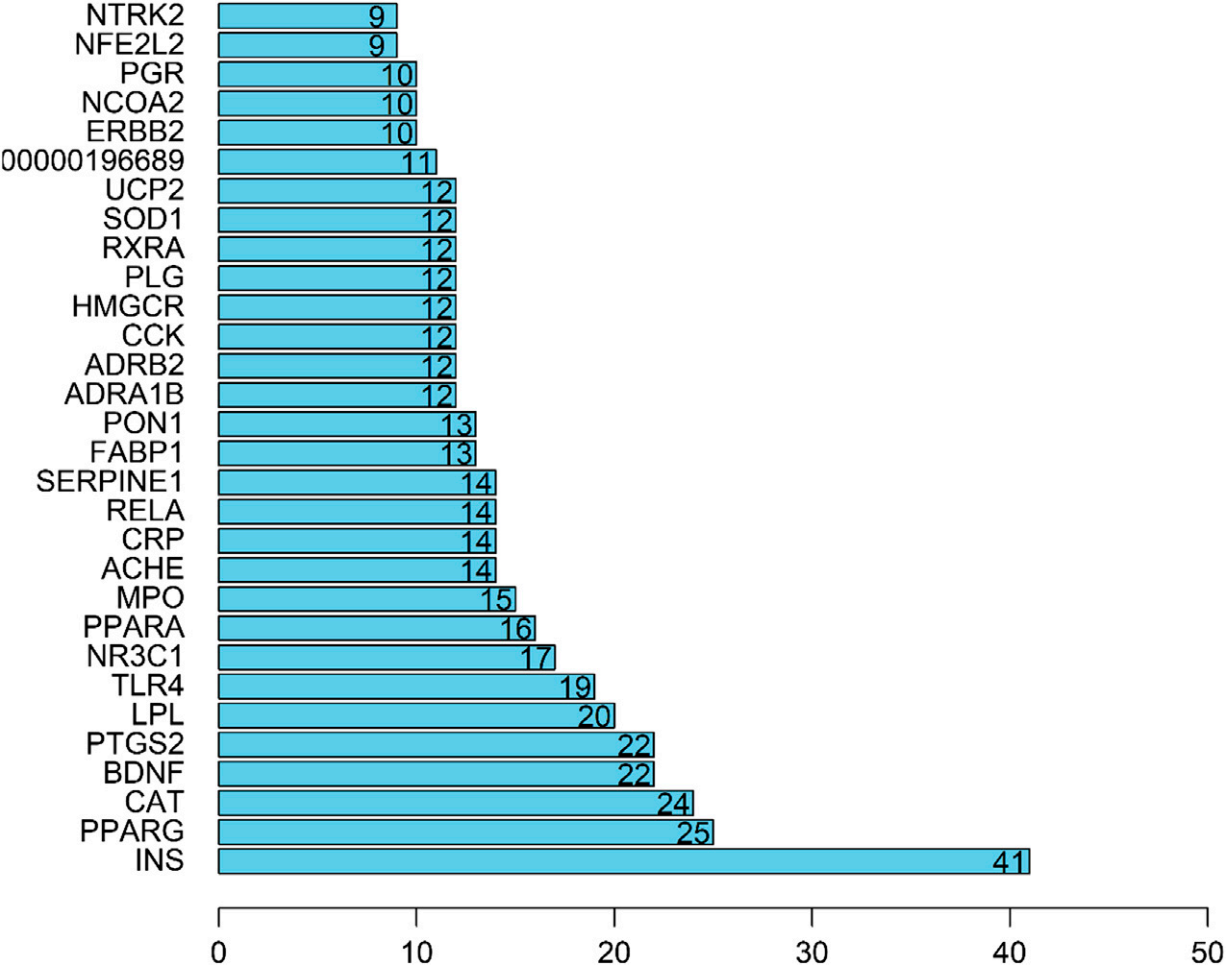
Core target of cinnamon for the treatment of breast cancer.

In the String database, the PPI network of the 61 targets was constructed. After two free nodes were deleted, a total of 59 nodes and 295 edges were found (Figure 3). Thereafter, we sorted the targets according to the number of connected nodes (Figure 4) to obtain the core targets, including insulin (INS), peroxisome proliferator-activated receptor gamma (PPARG), catalase (CAT), brain-derived neurotrophic factor (BDNF), and prostaglandin-endoperoxide synthase 2 (PTGS2).

### GO Biological Process and KEGG Pathway Enrichment Analysis

**FIGURE 5:**
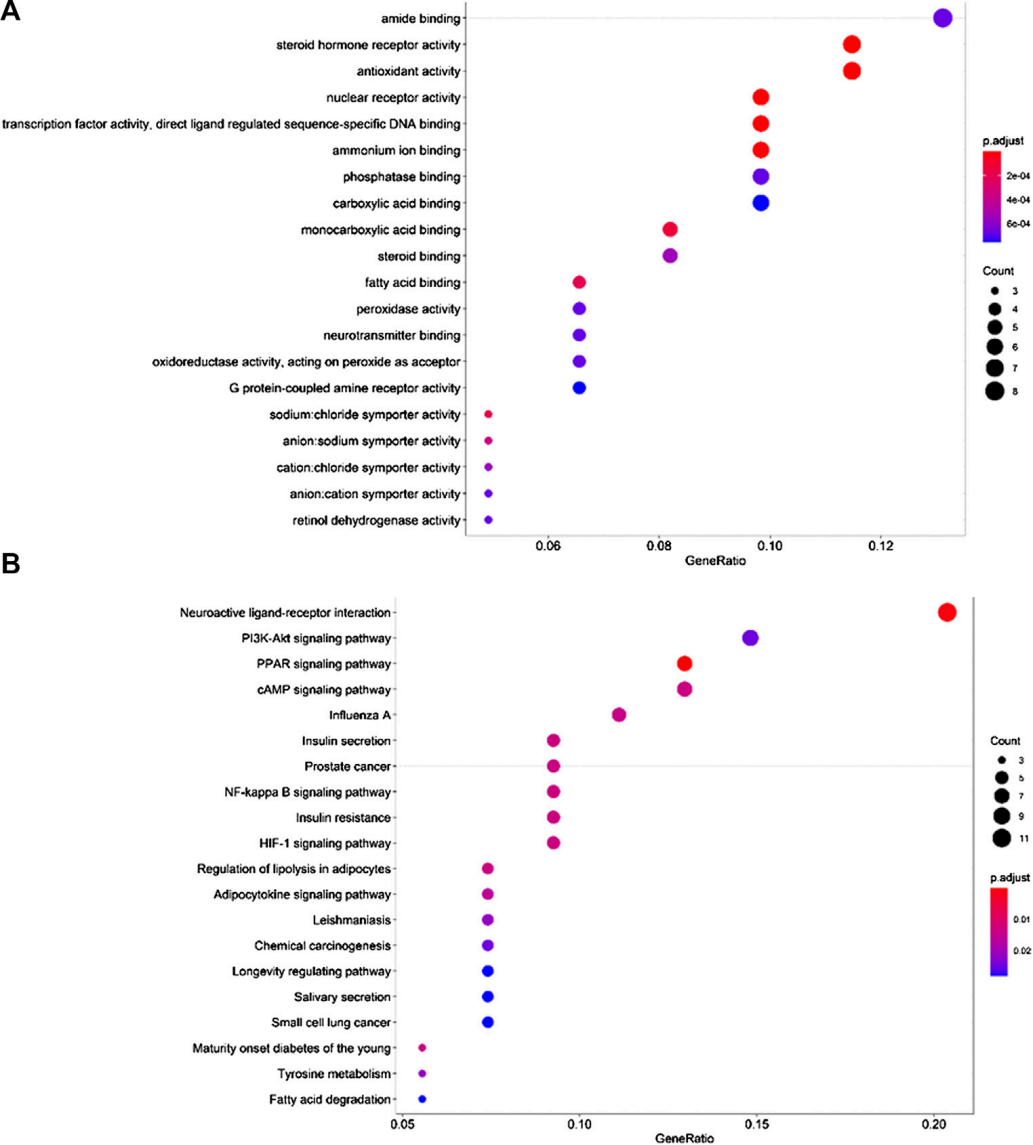
Top 20 GO terms and KEGG pathways. **(A)** GO biological process analysis, **(B)** KEGG pathway analysis.

Through GO function enrichment analysis, we obtained 83 items related to breast cancer, the top 20 of which include the following (Figure 5A): amide binding (GO:0033218), steroid hormone receptor activity (GO:0003707), antioxidant activity (GO:0016209), ammonium ion binding (GO:0070405), protease binding (GO:0002020), carboxylic acid binding (GO:0031406), monocarboxylic acid binding (GO:0033293), steroid binding (GO:0005496), fatty acid-binding (GO:0005504), peroxidase activity (GO:0004601), neurotransmitter binding (GO:0042165), oxidoreductase activity, G protein-coupled amine receptor activity (GO:0008227), sodium: chloride symporter activity (GO:0015378), anion: cation symporter activity (GO:0015296), and retinol dehydrogenase activity (GO:0004745). Therefore, it is speculated that cinnamon mainly exerts its anti-breast cancer effects through the above biological processes.

To further reveal the pathways employed by cinnamon against breast cancer, we used *p* < 0.05 as the screening criterion, conducted a KEGG pathway enrichment analysis on 59 targets, and screened out 37 pathways related to breast cancer. Accordingly, we listed the top 20 related items, including neuroactive ligand-receptor interaction (hsa04080), PI3K-Akt pathway (hsa04151), PPAR pathway (hsa03320), cAMP pathway (hsa04024), NF-kappa B pathway (hsa04064), and HIF-1 pathway (hsa04066) (Figure 5B). Based on these pathways, the anti-cancer effect of cinnamon on breast cancer may result from a complex multi-pathway synergetic effect.

### Inhibitory Effect of Cinnamaldehyde on the Growth of Breast Cancer Cells

**FIGURE 6:**
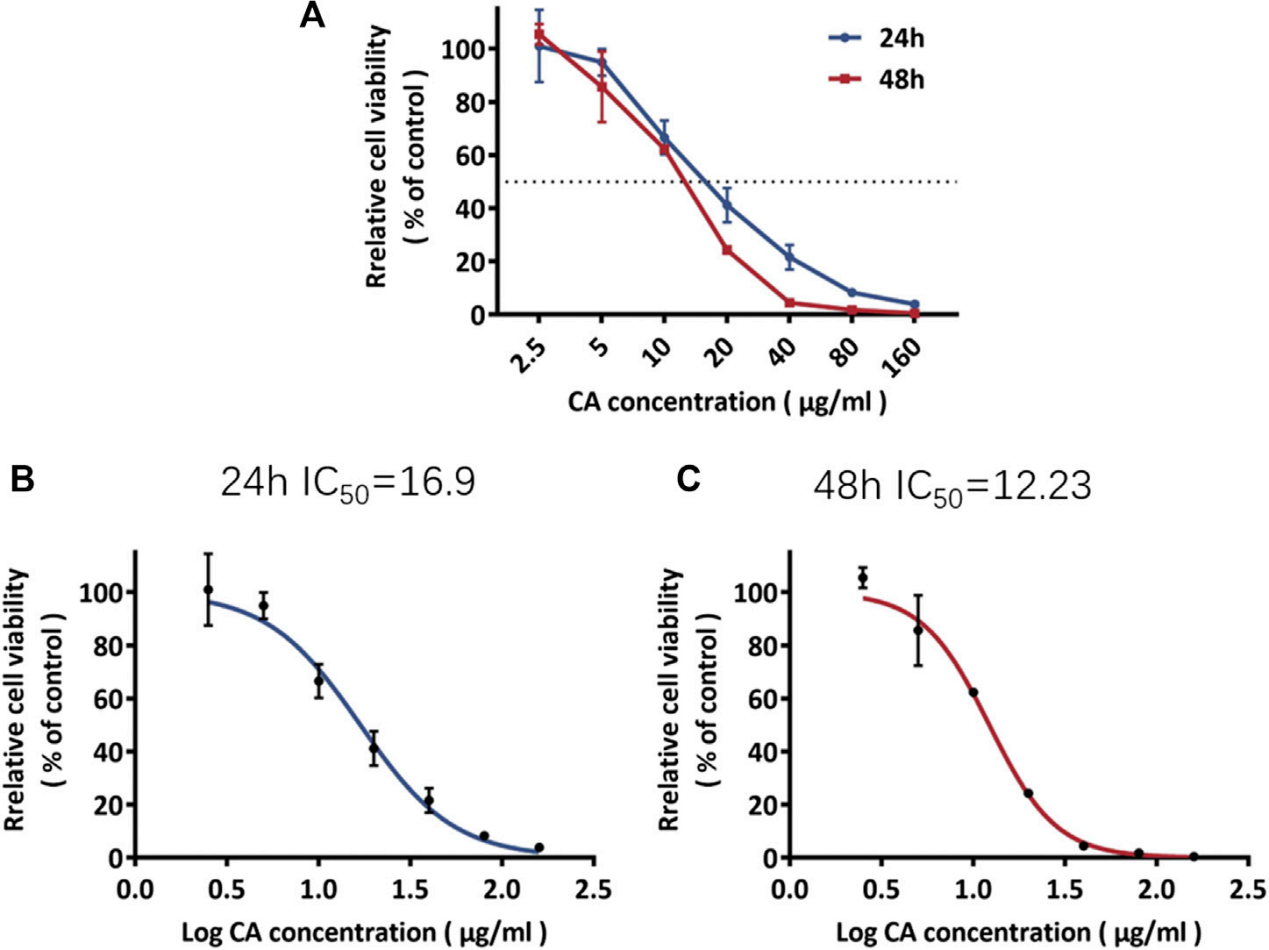
Rate of inhibition of MDA-MB-231 cell proliferation and the IC_50_ of cinnamaldehyde. CA, cinnamaldehyde.

MDA-MB-231 cells were treated with cinnamaldehyde. Our results indicated that 2.5, 5, 10, 20, 40 μg/ml cinnamaldehyde inhibited cell proliferation (Figure 6A). The IC_50_ of cinnamaldehyde at 24 and 48 h was 16.9 μg/ml and 12.23 μg/ml, respectively, with a 95% confidence interval of 17.81–44.20 (Figures 6B,C).

### Cinnamaldehyde Affects the Morphological and Cytoplasmic Changes of MDA-MB-231 Cells

**FIGURE 7:**
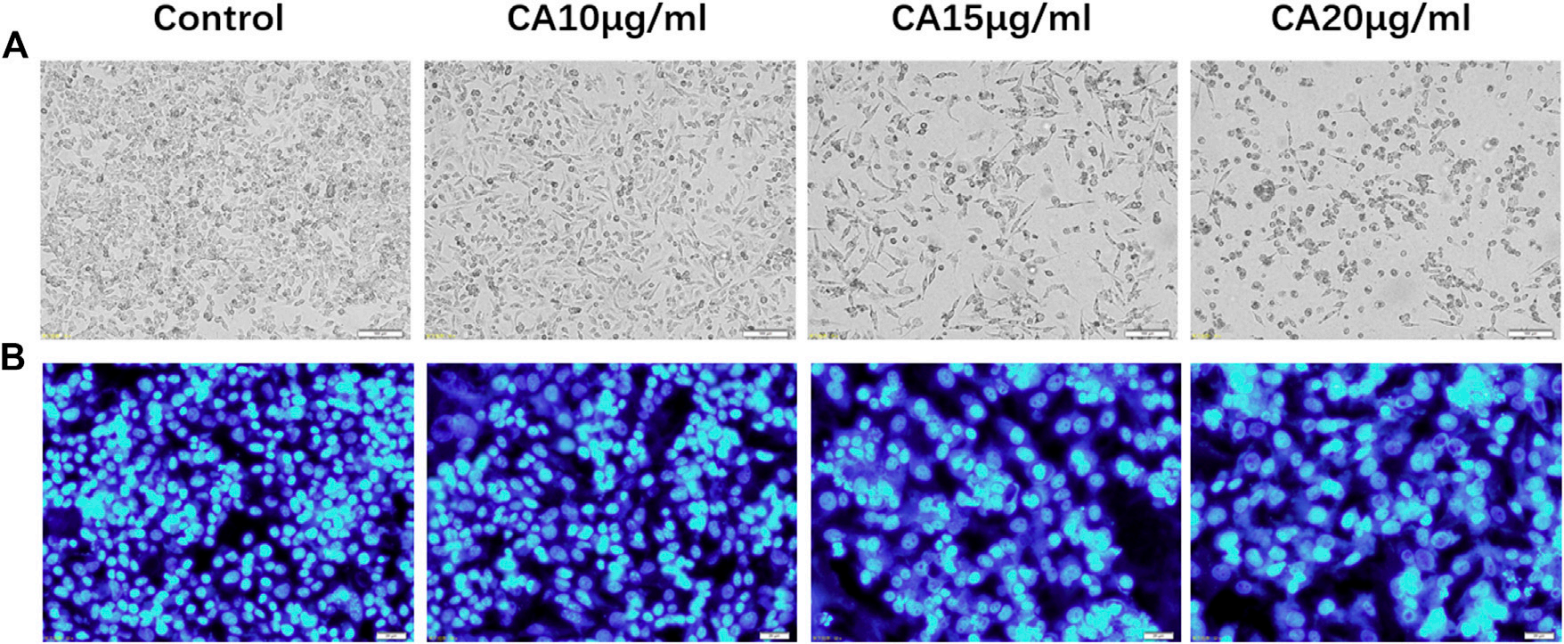
Cell morphological and cytoplasmic changes in MDA-MB-231 cells in the different treatment groups. **(A)** Cell morphological changes of MDA-MB-231 cells in different treatment groups (original magnification, ×100); **(B)** Hoechst 33,258 staining shows changes in the cytoplasm of MDA-MB-231 cells in different cinnamaldehyde treatment groups (original magnification, ×200). CA, cinnamaldehyde.

In the blank group, the MDA-MB-231 cells were fibrous, uniform in size, smooth, and refractive. Further, the cells exhibited normal growth. However, after 48 h of treatment with different concentrations of cinnamaldehyde (10, 15, 20, and μg/ml), the number of MDA-MB-231 cells significantly decreased, with the 20-μg/ml intervention group demonstrating a more evident decrease. Cinnamaldehyde inhibited the growth and proliferation of MDA-MB-231 cells. In addition, the characteristic morphology of the cells gradually disappeared, the number of cells decreased, the fibers became shorter, and some cells began to cluster (Figure 7A). Hoechst 33258 staining showed that with an increase in cinnamaldehyde concentration, the cell spacing of MDA-MB-231 became significantly larger, the cell morphology became rounder, the nucleus became larger, chromatin pyknosis was evident, and nuclear fragmentation was aggravated.

### Promoting Effect of Cinnamaldehyde on the Apoptosis of Breast Cancer Cells

**FIGURE 8:**
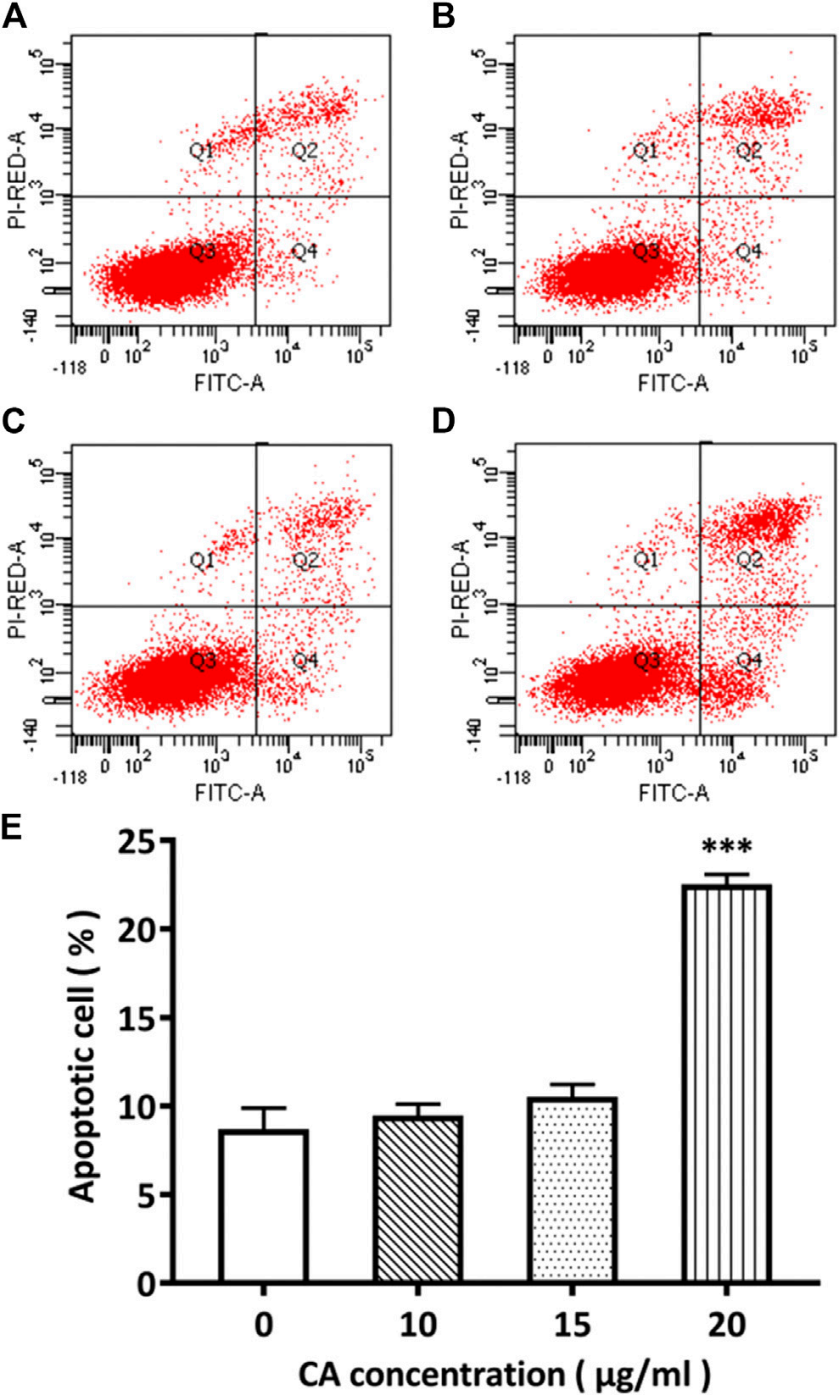
Comparison of the apoptotic rate of MDA-MB-231 cells administered different concentrations of cinnamaldehyde by Annexin V-FITC and PI double staining. **(A)**–**(E)** shows the cell apoptotic rate in the cinnamaldehyde groups administered different concentrations of cinnamaldehyde (∗∗∗compared to the control group *p* < 0.001). CA, cinnamaldehyde.

Flow cytometry was used to detect the effect of different concentrations of cinnamaldehyde on apoptosis. Our results showed that MDA-MB-231 cells exhibited 8.7, 9.5, 10.5, and 22.5% apoptosis when treated with 0, 10, 15, and 20 μg/ml of cinnamaldehyde, respectively. Compared with the control group, the cinnamaldehyde group in the normal quadrant of the 20-μg/ml cinnamaldehyde intervention group was significantly reduced, whereas the early apoptosis quadrant was significantly increased (*p* < 0.01; Figure 8).

### Inhibitory Effect of Cinnamaldehyde on the Migration and Invasion of Breast Cancer Cells

**FIGURE 9:**
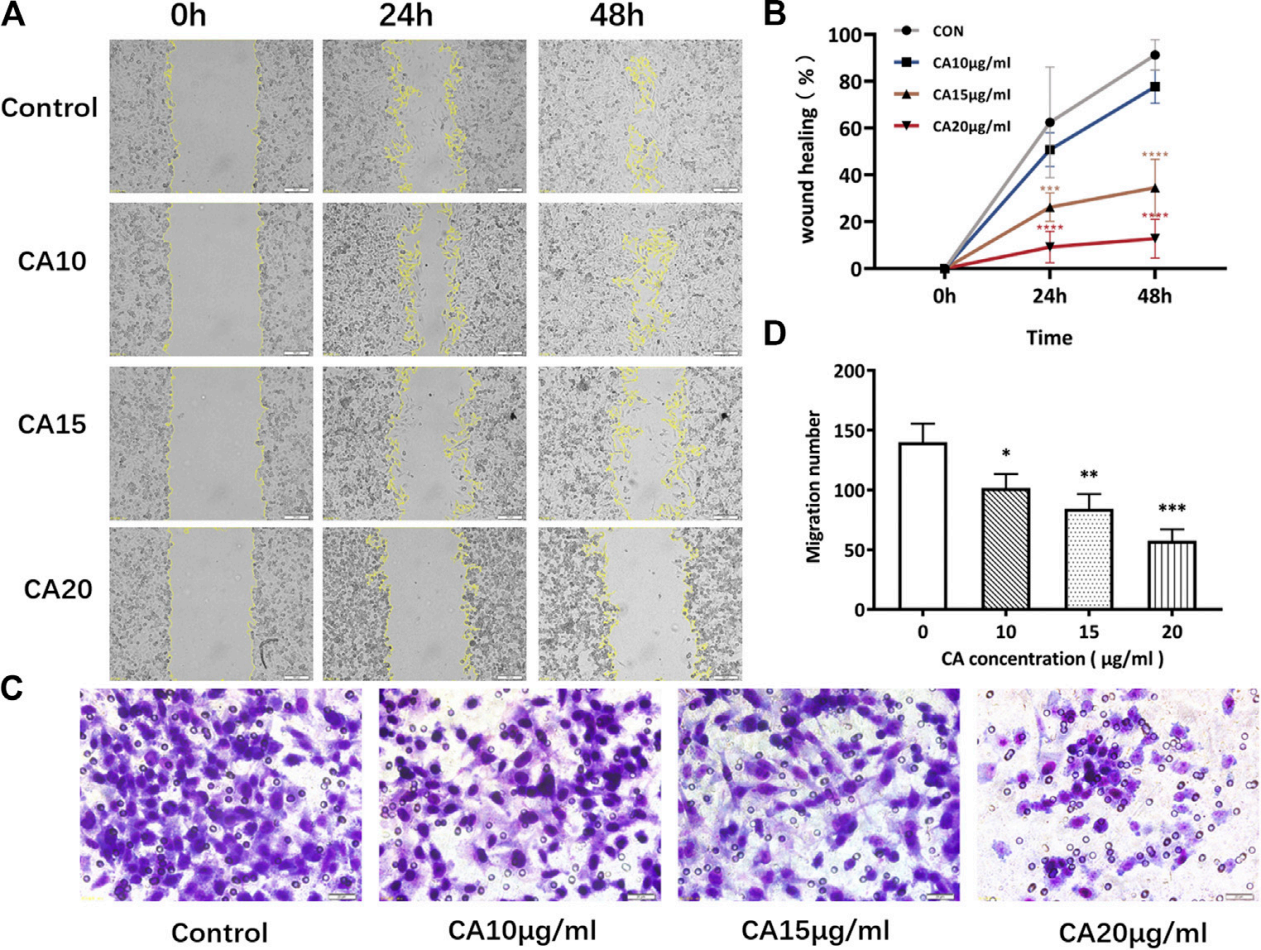
Effects of different concentrations of cinnamaldehyde on the migration and invasion ability of MDA-MB-231 cells after a 48-h intervention. **(A), (B)**, Wound healing assay of MDA-MB-231 cells in different treatment groups (original magnification, ×100). **(C), (D)**, Invasive assay after 48 h of CA intervention (original magnification, ×200) (∗, ∗∗, ∗∗∗, ∗∗∗∗ compared to the control group *p* < 0.05, *p* < 0.01, *p* < 0.001, and *p* < 0.0001, respectively). CA, cinnamaldehyde.

Transwell experiments revealed that MDA-MB-231 cells had a strong invasive ability. However, after 48 h of cinnamaldehyde intervention, the invasive ability of the cells was reduced in all intervention groups, especially in the 15 and 20 μg/ml groups (*p* < 0.01). In addition, the wound healing test results revealed that 15 and 20 μg/ml cinnamaldehyde intervention significantly reduced the migration ability of MDA-MB-231 cells (*p* < 0.05) (Figures 9A,B). Based on the transwell invasion test, the number of transmembrane cells in the control group was 159.3 ± 12.22, whereas that in the cinnamaldehyde group (5, 10, and 15 μg/ml) was 129.3 ± 25.11, 74.67 ± 16.17, and 56 ± 9.54, respectively. Our results showed that there was a statistically significant difference (*p* < 0.05) between the cinnamaldehyde group and the control group as well as a dose-effect relationship (Figures 9C,D). Such findings indicate that cinnamaldehyde could significantly inhibit the invasion of breast cancer MDA-MB-231 cells.

## Discussion

In recent years, network pharmacology has enabled the integration of functions such as high-throughput omics data analysis, virtual computer calculation, and network database retrieval. Therefore, it has been widely employed in research on a pharmacological basis and as a mechanism of action of Chinese medicine and traditional herbs (Yin et al., 2019; Li et al., 2020). Network pharmacology can combine system biology with multi-directional pharmacology, explore the correlation between the target of each component in traditional Chinese medicine preparations and diseases, and explain its mechanism of action (Zhang et al., 2019). The multi-component and multi-target network research model of network pharmacology breaks the traditional single-component, single-target research model, ultimately providing a new method for comprehensive analysis of compound mechanisms (Yuan et al., 2017). Existing studies have shown that in recent years, network pharmacology has been widely used in the screening of breast cancer and metastatic therapy drugs and targets (Yang et al., 2019; Mao et al., 2020).

In the present study, the effective components of the ancient herb, cinnamon, were analyzed through network pharmacology, and a network of cinnamon-chemical component-breast cancer targets was constructed. Based on our findings, the key chemical constituents in cinnamon were of 12 types, including EIC, oleic acid, DIBP, and cinnamaldehyde, corresponding to 66 active targets, including 61 common targets related to breast cancer. The PPI results showed that its key targets for breast cancer include PPARG, TLR-4, BDNF, and PPAR-α. PPARG is a nuclear receptor that is widely involved in the regulation of lipid metabolism, glucose homeostasis, and tumor progression due to its role as a transcription factor (Shen et al., 2020). Recent studies have shown that PPARG has a tumor suppressor effect and can inhibit the proliferation, migration, and invasion of breast cancer cells (Tan et al., 2013). Corresponding clinical studies have also confirmed that among breast cancer patients, those with high PPARG expression levels have a higher survival rate than patients with low PPARG expression levels (Xu et al., 2019). In this study, we found that the main compounds in cinnamon are most closely related to targets related to breast cancer cell apoptosis, invasion, and metastasis (Wang et al., 2016). For example, the upregulation of TLR-4 and PPAR-α expression is related to breast cancer cell apoptosis (Geng et al., 2018). Further, the upregulation of the adipose transcription factor, PPAR-α, can promote the migration and invasion of breast cancer cells (Blucher et al., 2020). The reduction of NCOA2 expression can induce breast cancer cell apoptosis by regulating the MAPK-ERK signaling pathway (Cai et al., 2019). The upregulation of BDNF can promote breast cancer cell proliferation and invasion (Gao et al., 2017). Therefore, we speculate that cinnamon and its main chemical components may exert an anti-breast cancer effect by regulating the targets and pathways related to breast cancer cell apoptosis, invasion, and metastasis.

GO analysis results showed that the key targets were involved in amide binding, steroid hormone receptor activity, antioxidant activity, transcription factor activity, direct ligand regulated sequence-specific DNA binding, carboxylic acid-binding, steroid binding, fatty acid-binding, peroxidase activity, neurotransmitter binding, oxidoreductase activity (acting on peroxide as an acceptor), G protein-coupled amine receptor activity, and other biological processes. Further pathway analysis revealed that cinnamaldehyde in cinnamon and breast cancer targets are mainly involved in the neuroactive ligand-receptor interaction, PI3K-Akt signaling pathway, PPAR signaling pathway, cAMP signaling pathway, NF-kappa B signaling pathway, and other pathways closely related to cancer. Cinnamaldehyde can also participate in the regulation of cell apoptosis, cell metabolism, inflammation, and other pathways.

The network pharmacological screening results revealed that cinnamaldehyde is one of the main active ingredients in cinnamon. Previous studies have shown that cinnamaldehyde and cinnamaldehyde-derived compounds are drug candidates for the development of anticancer drugs, which has attracted extensive research attention (Hong et al., 2016). Cinnamaldehyde can improve the anti-cancer efficacy of oxaliplatin by promoting the apoptosis of colorectal cancer cells *in vivo* and *in vitro* (Wu et al., 2019). As an antioxidant, cinnamaldehyde can inhibit the spread of cancer by inhibiting the expression of extracellular and intracellular fat factor nicotinamide phosphoribosyltransferase (Chiang et al., 2019). Although compared with other cancers, the role of cinnamaldehyde in breast cancer is not well understood. However, in recent years, related studies have also discovered the anti-proliferation effect of cinnamaldehyde on breast cancer cells *in vitro* and *in vivo* (Lu et al., 2010). In this study, through network pharmacology, we predicted that the targets related to the anti-breast cancer effect of cinnamaldehyde are mainly associated with breast cancer cell apoptosis, invasion, and metastasis. However, the bioinformatics data used for target prediction can only reflect the correlation. Thus, its specific role in regulating breast cancer cell apoptosis, invasion, and metastasis remains unknown. As a result, we verified the effect of cinnamaldehyde on human breast cancer cells through cell experiments and further confirmed the anti-cancer effect and mechanism of the active ingredients of cinnamon. Our research is a supplement to previous research and further reveals the anti-breast cancer effect of cinnamaldehyde.

TNBC is an aggressive breast cancer subtype and one of the most clinically malignant breast cancers; however, there is currently a lack of targeted treatment options (Rigiracciolo et al., 2020). Herein, we selected human TNBC MDA-MB-231 cells for subsequent verification experiments. Impaired apoptosis plays a critical role in the initiation and progression of cancer (Sadeghi et al., 2019). Therefore, we speculated that as a therapeutic agent, cinnamaldehyde may exert an anti-breast cancer effect by affecting the apoptosis-related pathways of cancer cells. Our cell experiments demonstrated that cinnamaldehyde inhibited the proliferation of MDA-MB-231 cells, changed the cytoplasmic morphology, promoted the apoptosis of MDA-MB-231 cells, reduced the invasion and migration ability of MDA-MB-231 cells, and exhibited anti-breast cancer effects. The anti-breast cancer effect of cinnamaldehyde may be related to the eight targets selected for breast cancer. In future experiments, we will conduct further studies on its role and function.

**FIGURE 10:**
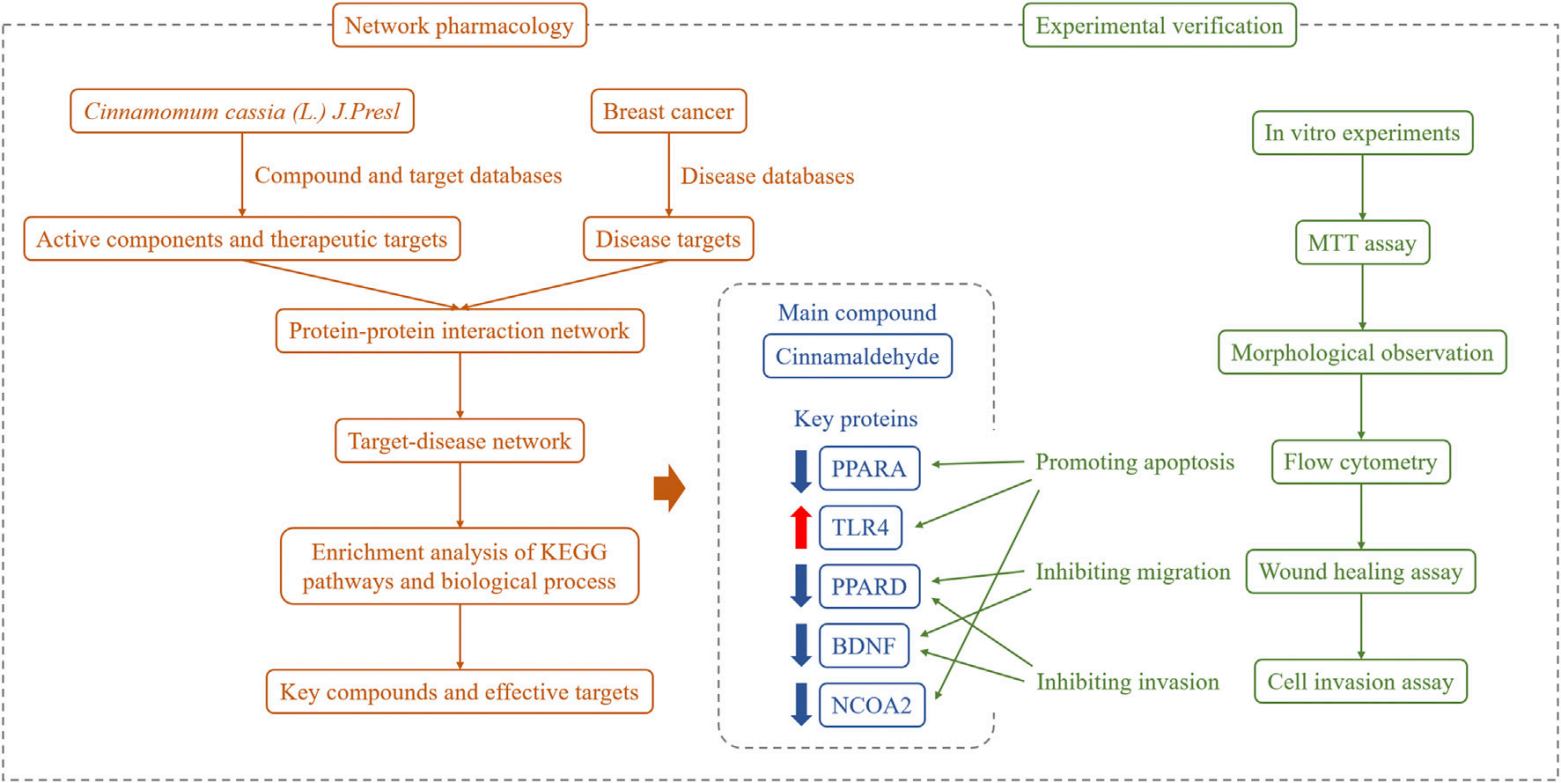
Overall framework of cinnamaldehyde for the treatment of breast cancer. Through the network pharmacological integration strategy, cinnamaldehyde (the main chemical component of cinnamon in breast cancer) was selected. Based on *in vitro* cell experiments, cinnamaldehyde was confirmed to promote the apoptosis of breast cancer tumor cells and inhibit cell migration and invasion.

In summary, in the present study, we revealed the main active ingredient in cinnamon and explored its potential targets for the treatment of breast cancer. By establishing a breast cancer disease network and enriching the key nodes and pathways for the regulation of cinnamon active ingredients, we found that antioxidant activity and the PI3K-Akt and NF-κB signaling pathways play important roles in the pharmacological effects of cinnamon. Furthermore, through breast cancer cell experiments, we confirmed that cinnamaldehyde, the main anti-breast cancer component in cinnamon, can inhibit cell proliferation, invasion, and migration; change cytoplasmic morphology; and promote apoptosis. Our research findings provide an experimental and theoretical basis for further applications of cinnamaldehyde in the treatment of breast cancer (Figure 10).

## Data Availability Statement

The original contributions presented in the study are included in the article/Supplementary Material, further inquiries can be directed to the corresponding author.

## Author Contributions

XP designed the experiments; YL and TA performed the experiments and wrote the manuscript. BY, DW, and YF analyzed the data. All authors reviewed the manuscript.

## Funding

This work was financially supported by grants from the National Natural Science Foundation of China (NSFC81774319), Beijing Natural Science Foundation Project (7182098), and the independent subject graduate student projects of Beijing University of Traditional Chinese Medicine (2019-JYB-XS).

## Conflict of Interest

The authors declare that the research was conducted in the absence of any commercial or financial relationships that could be construed as a potential conflict of interest.

## Glossary

cinnamon: *Cinnamomum cassia* (L.) J. Presl
TNBC: Triple-negative breast cancer
TCMSP: Traditional Chinese Medicine Systems Pharmacology Database and Analysis Platform
DL: Drug-likeness
TCM: Traditional Chinese Medicine
TCMID: The Traditional Chinese Medicines Integrated Database
FDR: False discovery rate
OB: Oral bioavailability
PPI: Protein–protein interaction
TS: Tanimoto similarity
STP: Swiss Target Prediction
OMIM: Online Mendelian Inheritance in Man
DAVID: The Database for Annotation, Visualization, and Integrated Discovery
GO: gene ontology
KEGG: Kyoto encyclopedia of genes and genomes
OD: optical density
FITC: fluorescein isothiocyanate
PI: Propidium iodide
SD: standard deviation
PPARG: Peroxisome Proliferator-Activated Receptor Gamma
INS: Insulin
BDNF: Brain-derived neurotrophic factor
CAT: catalase
CA: Cinnamaldehyde
