# Supplementary figures and images for: Targets and Mechanism Used by Cinnamaldehyde, the Main Active Ingredient in Cinnamon, in the Treatment of Breast Cancer
Source: Front Pharmacol. 2020 Dec 9;11:582719. doi: 10.3389/fphar.2020.582719 (PMC7848847; doi:10.3389/fphar.2020.582719)

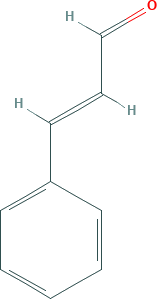

Supplement: Supplementary file 1 [file Image1.tif]
